# Differing associations with childhood outcomes using behavioural patterns derived from three data reduction techniques

**DOI:** 10.1093/ije/dyac142

**Published:** 2022-07-13

**Authors:** Ninoshka J D’Souza, Miaobing Zheng, Gavin Abbott, Sandrine Lioret, Kylie D Hesketh

**Affiliations:** Institute for Physical Activity and Nutrition, School of Exercise and Nutrition Sciences, Deakin University, Burwood, VIC, Australia; Institute for Physical Activity and Nutrition, School of Exercise and Nutrition Sciences, Deakin University, Burwood, VIC, Australia; Institute for Physical Activity and Nutrition, School of Exercise and Nutrition Sciences, Deakin University, Burwood, VIC, Australia; Research Center in Epidemiology and Biostatistics, Université de Paris, INSERM, INRA, Paris, France; Institute for Physical Activity and Nutrition, School of Exercise and Nutrition Sciences, Deakin University, Burwood, VIC, Australia

**Keywords:** Children, principal component analysis, cluster analysis, latent profile analysis, health, overweight, obesity, health-related quality of life, academics

## Abstract

**Background:**

Behavioural patterns help to understand the influence of multiple health behaviours on childhood outcomes. Behavioural patterns derived using different data reduction techniques can be non-identical and may differentially associate with childhood outcomes. This study aimed to compare associations of behavioural patterns derived from three methods with three childhood outcomes.

**Methods:**

Data were from the Healthy Active Preschool and Primary Years study when children were 6–8 years old (*n* = 432). Cluster analysis (CA), latent profile analysis (LPA) and principal component analysis (PCA) were used to derive behavioural patterns from children’s diet, physical activity, sedentary behaviour and sleep data. Behavioural data were obtained through parent report and accelerometry. Children’s height, weight and waist circumference were measured by trained study staff. Health-related quality of life data were obtained using the Pediatric Quality of Life Inventory and academic performance scores were from a national test. Associations between derived patterns from each method and each of the outcomes were tested using linear regression (adjusted for child age and sex and parent education).

**Results:**

Three patterns were each derived using CA and LPA, and four patterns were derived using PCA. Each method identified a healthy, an unhealthy and a mixed (comprising healthy and unhealthy behaviours together) pattern. Differences in associations were observed between pattern groups from CA and LPA and pattern scores from PCA with the three outcomes.

**Conclusions:**

Discrepancies in associations across pattern derivation methods suggests that the choice of method can influence subsequent associations with outcomes. This has implications for comparison across studies that have employed different methods.

Key Messages

- Behavioural patterns help to understand the combined influence of multiple health behaviours on childhood outcomes.
- There exist multiple data reduction methods to identify behavioural patterns.
- Behavioural patterns derived using three different data reduction techniques identified non-identical patterns and were found to associate differentially with three childhood outcomes.
- The choice of method to derive behavioural patterns can influence associations with childhood outcomes.
- Comparisons of findings from studies using different methods to derive behavioural patterns may not be appropriate and must be done with caution.

## Introduction

Owing to the aetiology of most outcomes being complex and multifactorial in nature, studies examining childhood outcomes have shifted towards the investigation of multiple health behaviours.^1^ Multiple behaviours that influence outcomes can be collectively examined using data reduction techniques which generate patterns of these behaviours, to test for subsequent associations with health outcomes.^1–3^ These methods are advantageous, as they overcome the limitations of examining behaviours individually which do not account for the synergistic effects of multiple health behaviours on a particular outcome or are prone to multicollinearity in multivariable models.^4^

Given that different data reduction techniques are based on differing statistical algorithms, the patterns derived across methods may not be identical within a single dataset.^5^ The resultant pattern outputs also vary across methods, with some producing continuous scores for each individual for each identified pattern [e.g. principal component analysis (PCA)], whereas other methods [e.g. cluster analysis (CA) or latent profile analysis (LPA)] identify distinct groups (clusters) of individuals displaying similar patterns within the whole study sample. Furthermore, patterns derived using different techniques across studies are not directly comparable as these methods are data driven, making the patterns derived specific to a particular sample. Therefore, comparisons are more appropriate within a single dataset. Our previous analysis deriving patterns using three techniques (CA, LPA and PCA) in a single dataset^5^ found discrepant patterns across methods with some concordance in pattern characteristics. It is logical to posit that these resulting differential patterns might affect subsequent associations with childhood outcomes.

A limited number of studies have investigated comparisons of associations of dietary patterns (derived using different techniques) and health outcomes. The majority of studies was in adults,^6–11^ with one study in toddlers.^12^ All studies assessed associations of patterns with a single health outcome. Outcomes included cardiovascular risk,^6^^,^^8^^,^^10^ colorectal cancer,^9^ depression^11^ and bone health^7^ for adults and obesity in toddlers. To our knowledge, only one study investigated comparisons of associations between patterns comprising two behaviours (physical activity and sedentary behaviour) and overweight in adolescents.^13^ Most studies reported dissimilar numbers of patterns derived across methods compared. Both similarities and differences in associations with patterns across methods were reported, suggesting varying results are possible using different methods. The evidence from these studies, having investigated only one (e.g. diet) or two behaviour domains, does not provide a comprehensive picture of the synergistic effects of multiple behaviours as behavioural patterns on health outcomes. No studies assessing comparisons of multi-behavioural patterns identified using different methods and associations with outcomes in school-aged children have been published. Childhood outcomes encompass an exhaustive list; however, the present study focused on three important outcomes: childhood overweight and obesity, health-related quality of life (HRQoL) and academic performance. Three commonly used data reduction techniques (CA, LPA and PCA) were used to derive behavioural patterns.^1^

Therefore, extending our previous analysis, this study aimed to compare associations between patterns (from four behaviour domains: diet, physical activity, sedentary behaviour and sleep) using CA, LPA and PCA and three childhood outcomes (adiposity, HRQoL and academic performance) in a single dataset of Australian children aged 6–8 years.

## Methods

### Study setting and participants

Data from the second wave (T2) of the Healthy Active Preschool and Primary Years (HAPPY) study were used. The study has been previously described in detail.^14^ Briefly, the sample began with a cohort of 1002 parents of 3–5-year-old children in 2008/09. Children were followed up at two time points, in 2011/12 and 2012/13. The Deakin University Human Research Ethics Committee (EC 291–2007), the Department of Education and Early Childhood Development (2011_001008) and the Catholic Education Office (1714) granted ethical approval for the study. Parents provided written informed consent at all time points to participation. Data were captured using a parent-report survey and accelerometers at T2. Key measures are described below.

### Exposure variables

#### Parent survey

Dietary data were obtained using a validated food frequency questionnaire.^15^ The parent survey included 15 items that reported sufficient reliability, to capture intake frequencies of fruit, vegetables and discretionary food items.^15^ A 7-point scale (0–6 or more times) was used to record the frequency of consumption of discretionary food items in the previous week. These were categorized into six sweet and seven savoury discretionary items, which were first summed and then divided by seven to obtain daily intake values. A 6-point scale (0–5 or more times) captured the intake frequency of fruits and vegetables, respectively, in the past 24 h and represented daily consumption.

Children’s time spent in organized sports, outdoor play, screen viewing, videogames, quiet play and sleep were recorded using the parent survey (in hours and minutes).^16^ Parents reported the total duration their children spent in football, basketball, soccer, swimming, netball, gymnastics, dance, cricket and ‘other’ sports, in a given week. Weekly total duration for each sport was summed to obtain total weekly organized sport duration, and then divided by seven to obtain daily equivalents. Time children spent playing outdoors were reported for a typical weekday and a weekend day. Average day duration was obtained as a weighted score, whereby weekday and weekend day durations over five and two days, respectively, were summed together and then divided by seven. Total time children spent on the following sedentary behaviours: screen time (television viewing + computer use excluding games), videogames (computer + handheld electronic games) and quiet playtime during the week (Monday–Friday) and the weekend (Saturday–Sunday) were also reported. Sums of total weekday and weekend durations for each activity, divided by seven, provided daily durations. Children’s usual nightly sleep duration was reported in hours and minutes to provide daily sleep duration. All measures reported sufficient reliability.^5^

#### Accelerometry

Physical activity and sedentary behaviour were additionally measured objectively using Actigraph GT1M uniaxial accelerometers (Pensacola, FL, USA). Accelerometers were fitted on children in person, were hip-worn for eight consecutive days during waking hours and removed for water based activities. Accelerometer data were captured in 15-s epochs, and if recorded for a minimum of 8 h a day for ≥4 days, including one weekend day, were considered valid. Non-wear time was defined as consecutive zero counts ≥10 min. A total of 534 children wore accelerometers, 445 had valid data (three monitors were lost, two failed and 84 children had invalid data). Accelerometer data with counts >2296/min and counts <100/min were classified as moderate-to-vigorous physical activity (MVPA) and sedentary time (ST), respectively.^17^ Residuals obtained by regressing accelerometer data on wear time were used to adjust MVPA and ST to total wear time.

### Outcome variables

#### Adiposity

Children’s height (using a Wedderburn Seca portable rigid stadiometer), weight (using a Wedderburn Tanita digital portable scale) and waist circumference (using a steel non-stretch tape) were measured by trained study staff using standardized protocols. Measurements were taken twice, either at school or at home. If there was a discrepancy between the first two recorded measurements (weight >0.5 kg, height >0.5 cm, waist circumference >0.1 cm) a third measurement was taken. The final value was recorded as the average of the two closest measurements.^18^ Height and weight were used to calculate body mass index (BMI), and further converted using International Obesity Task Force (IOTF) cut-offs, into age and sex-specific BMI z-scores.^19^ These cut points were used to classify children’s weight status (underweight or healthy, overweight, and obese).^19^^,^^20^

#### Health-related quality of life

Children’s HRQoL was assessed using the previously validated 15-item Pediatric Quality of Life Inventory (PedsQL).^21^ Parents reported their children’s (*n* = 224) emotional, social and school functioning (five items each) using a 5-point Likert scale (never = 0, almost never = 1, sometimes = 2, often = 3, almost always = 4). Individual item scores for each domain were first reversed scored (0 = 100, 1 = 75, 2 = 50, 3 = 25, 4 = 0), summed and then averaged to obtain emotional, social and school functioning scores*.* Higher scores indicate higher domain functioning. These three scores were summed and averaged to obtain a psychosocial functioning score.

#### Academic performance

Results of the Year 3, National Assessment Program—Literacy and Numeracy (NAPLAN) test were used to obtain scores for five academic domains: reading, writing, spelling, numeracy and language (grammar and punctuation).^22^ Academic scores were converted to scale scores ranging 0–1000 (with a mean of 500 and standard deviation of 100). Parents provided consent for release of NAPLAN results from the Australian Curriculum Assessment and Reporting Authority (ACARA). Scores (*n *= 348) were matched to the HAPPY study participants.

### Confounders

Child age and sex and parental education (through parent-report survey) were included as confounders for each of the outcome variables. Parent (or main carer) education level was grouped into university and non-university education.

### Data analysis

Analyses were conducted using Stata 16.0 (StataCorp, TX, USA) and Mplus 8.0. Behavioural patterns were derived using three methods: CA, LPA and PCA. All three methods included 12 input variables (four diet: fruit, vegetable, sweet and savoury discretionary items; three physical activity: organized sport, outdoor play duration and MVPA levels; four sedentary behaviour: screen, videogame, quiet play and ST; and one sleep variable: sleep duration). For use in data reduction models, these input variables were standardized with means of 0 and standard deviations of 1 due to inconsistency of scales capturing behavioural data.

Pattern derivation analyses using CA, LPA and PCA within the same cohort have been previously described in detail.^5^ The patterns derived using CA and LPA represent mutually exclusive groups of children displaying distinct patterns whereas the patterns derived using PCA represent scores of each individual for each pattern. K-means CA alongside application of the Calinski–Harabasz criteria revealed the three-cluster model to be optimal. For LPA, a range of 2–10 pattern solutions were derived and compared against the adjusted Lo–Mendel–Rubin test, identifying the three-pattern model to be optimal and most interpretable. As behaviours can feature in multiple patterns across the three methods, a cut point of ±0.2 for the estimated standardized behavioural scores was used to differentiate behaviours being high or low for given patterns, and patterns were labelled accordingly. PCA, using Horn’s parallel analysis, revealed that the first four components were best to retain. Varimax rotation was applied to improve component interpretation. In brief, each method identified healthy, unhealthy and mixed patterns. Patterns derived from each method are described in Table 1. The distribution of children into different pattern groups across LPA and CA is provided in Supplementary Table S1 (available as Supplementary data at *IJE* online). For LPA and CA, there was some concordance in the number of children classified into the same pattern type (% agreement = 76.2%, kappa = 0.64, see Supplementary Table S2, available as Supplementary data at *IJE* online).

**Table 1:** Description of pattern groups identified using cluster analysis and latent profile analysis and pattern scores using principal component analysis^5^

Method	Unhealthy pattern group/scores	Healthy pattern group/scores	Mixed pattern group/scores
Cluster Analysis (CA)	1. Unhealthy (↓ F, ↓ V, ↓ OPA, ↑ SC, ↑ VG, ↑ ST)	2. Active healthy eaters (↑ F, ↑ V, ↑ OP, ↓ DFI, ↓ SC)	3. Active sleepers, non-sedentary unhealthy eaters (↑ MVPA, ↑ SL, ↓ F, ↓ VG, ↓ ST)
Latent Profile Analysis (LPA)	1. Unhealthy (↓ F, ↓ V, ↓ OP, ↓ MVPA, ↑ ST)	2. Active healthy eaters (↑ F, ↑ V, ↑ OP)	3. Active non-sedentary unhealthy eaters (↑ SDFI, ↑ MVPA, ↑ OP, ↓ F, ↓ ST)
Principal Component Analysis (PCA)^a^	1. Low sleep, sedentary, high snacks pattern score (↑ SDFI, ↑ SC, ↑ VG ↓ SL)	2. Active healthy eating pattern score (↑ F, ↑ V, ↑ OP)	3. Active, high sleep, non-sedentary, unhealthy eating pattern score (↑ DFI, ↑ MVPA, ↑ SL, ↓ ST) 4. Inactive, sedentary, high sleep pattern score (↑ QP, ↑ SL, ↓ OS, ↓ OP)

↓, lower; F, fruit; V, vegetables; OPA, overall physical activity; ↑, higher; SC, screen time; VG, videogames; ST, sedentary time; OP, outdoor play; DFI, discretionary food items; MVPA, moderate-to-vigorous intensity physical activity; SL, sleep; SDFI, savoury discretionary food items; QP, quiet play; OS, organized sports.

a The patterns have been re-labelled to reflect pattern scores. The total percentage variance explained by the four PCA components was 54% (component 1 = 16%, components 2 and 3 = 14% each, component 4 = 10%).

Cross-sectional associations between the derived patterns (exposure variables) and continuous outcomes [(i) adiposity (BMI, kg/m^2^ and waist circumference, cm), (ii) HRQoL (emotional, social, school, and psychosocial functioning scores), and (iii) academic performance (reading, writing, spelling, numeracy, and grammar scores)] were assessed using linear regression. Patterns derived using CA and LPA were included as categorical independent variables, whereas patterns (varimax rotated) derived using PCA, being continuous (representing scores), were included as independent variables in the regression models. For PCA, all patterns were included in the model to reflect adjustment for other patterns. Associations with patterns from CA and LPA describe differences in outcomes between pattern groups, whereas PCA describes associations of individual pattern scores with outcomes. Models were adjusted for child age and sex and parent education, and were fitted with cluster robust standard errors to account for potential clustering by recruitment centre. No adjustment was made for multiple testing, due to the exploratory nature of the study.^23^

## Results

Complete behavioural data were available for 432 children, to derive lifestyle patterns. Table 2 depicts the sample characteristics.

**Table 2:** Sample characteristics

Variable	*N*	Mean ± SD (range)
Child age (years)	432	7.6 ± 0.7 (5.4–9.1)
Sex [*n* (%)]	432	
Male		244 (56.5)
Female		188 (43.5)
Parent education	432	
Below university		144 (33.3)
University and above		288 (66.7)
Adiposity measures	432	
BMI (kg/m^2^)		16.5 ± 1.8 (12.9–25.4)
Waist circumference (cm)		58.6 ± 6.0 (27.9–85.0)
Health-related quality of life^a^	224	
Emotional functioning score		72.3 ± 15.1 (30–100)
Social functioning score		83.1 ± 15.9 (20–100)
School functioning score		81.2 ± 15.3 (31–100)
Psychosocial functioning score		78.9 ± 12.6 (27–100)
NAPLAN scores^a^	348	
Reading		479.6 ± 58.1 (94.5–617.6)
Writing		445.2 ± 58.1 (94.5–617.6)
Spelling		441.8 ± 76.5 (239.5–638.8)
Numeracy		450.9 ± 77.7 (232.1–740.6)
Grammar		484.3 ± 92.7 (193.0–771.5)

BMI, body mass index; SD, standard deviation; NAPLAN, National Assessment Program—Literacy and Numeracy testing.

a Higher scores indicate higher health-related quality of life and academic performance.

Associations of behavioural patterns, derived using CA, LPA and PCA, with childhood adiposity are presented in Table 3. There were no differences in adiposity measures between pattern groups derived using CA and LPA with adiposity measures. For PCA, the unhealthy, ‘low sleep, sedentary, high snacks’ pattern score was associated with higher BMI z-scores (0.09 units higher) and waist circumference (0.07 units higher); and the mixed ‘inactive, sedentary, high sleep’ pattern score was associated with lower BMI z-scores (0.1 unit lower). The mixed ‘active, high sleep, non-sedentary, unhealthy eating’ and the healthy ‘active healthy eating’ pattern scores were not associated with adiposity.

**Table 3:** Associations of behavioural patterns with adiposity at 6–8 years

Method	Behaviour pattern groups (CA/LPA)/scores (PCA)^b^	BMI z-score		Waist circumference	
		β (95% CI)	*P*-value	β (95% CI)	*P*-value
CA^a^	Active healthy eaters pattern group vs unhealthy pattern group	−0.07 (−0.36, 0.22)	0.64	−0.08 (−0.39, 0.23)	0.61
	Active sleepers, non-sedentary, unhealthy eaters pattern group vs unhealthy pattern group	−0.12 (−0.34, 0.11)	0.30	−0.20 (−0.48, 0.08)	0.16
	Active healthy eaters pattern group vs active, sleepers, non-sedentary, unhealthy eaters pattern group	−0.05 (−0.25, 0.15)	0.63	−0.12 (−0.36, 0.12)	0.32
LPA^a^	Active healthy eaters pattern group vs unhealthy pattern group	−0.05 (−0.28, 0.18)	0.70	0.00 (−0.24, 0.25)	0.97
	Active, non-sedentary, unhealthy eaters pattern group vs unhealthy pattern group	0.04 (−0.17, 0.25)	0.70	−0.00 (−0.24, 0.23)	0.97
	Active healthy eaters pattern group vs active non-sedentary, unhealthy eaters pattern group	0.09 (−0.15, 0.32)	0.47	−0.01 (−0.26, 0.24)	0.95
PCA^b^	‘Mixed’ active, high sleep, non-sedentary, unhealthy eating pattern score	−0.01 (−0.08, 0.06)	0.73	−0.04 (−0.13, 0.05)	0.38
	‘Healthy’, active healthy eating pattern score	0.02 (−0.05, 0.09)	0.56	0.06 (−0.02, 0.13)	0.14
	‘Unhealthy’, low sleep, sedentary, high snacks pattern score	0.09 (0.02, 0.16)	**0.015**	0.07 (0.01, 0.14)	**0.028**
	‘Mixed’ inactive, sedentary, high sleep pattern score	−0.10 (−0.19, −0.02)	**0.021**	−0.03 (−0.13, 0.07)	0.53

BMI, body mass index; CI, confidence interval; CA, cluster analysis; LPA, latent profile analysis; PCA, principal component analysis.

Bold values indicate significant *P-*values.

a Pairwise comparisons for patterns derived using CA and LPA.

b Patterns identified by PCA comprise behavioural scores for all sample members, patterns from CA and LPA comprise groups of individuals.

Associations between behavioural patterns and HRQoL are presented in Table 4. For CA, children in the mixed ‘active sleepers non-sedentary, unhealthy eaters’ pattern group had higher (5.7–7.5 units higher) estimated average scores for emotional, social,and psychosocial functioning, respectively, compared with children in the unhealthy pattern group. There were no differences in HRQoL scores between other pattern groups derived from CA. Similarly, no differences in HRQoL scores were found among pattern groups derived from LPA. For PCA, the mixed ‘active, high sleep, non-sedentary, unhealthy eating’ pattern score was associated with higher social functioning (1.8 units higher). Other pattern scores derived from PCA were not associated with any of the HRQoL outcomes.

**Table 4:** Associations of behavioural patterns with health related quality of life

Method	Behaviour pattern groups (CA/LPA)/scores (PCA)^b^	Emotional functioning		Social functioning		School functioning		Psychosocial functioning	
		β (95% CI)	*P*-value	β (95% CI)	*P*-value	β (95% CI)	*P*-value	β (95% CI)	*P*-value
CA^a^	Active healthy eaters pattern group vs unhealthy pattern group	5.43 (−0.36, 11.23)	0.066	4.77 (−2.03, 11.58)	0.17	1.82 (−4.26, 7.91)	0.55	4.00 (−1.41, 9.41)	0.15
	Active sleepers, non−sedentary, unhealthy eaters pattern group vs unhealthy pattern group	6.02 (0.96, 11.08)	**0.021**	7.50 (1.39, 13.60)	**0.017**	3.60 (−3.26, 10.5)	0.30	5.70 (0.41, 11.00)	**0.035**
	Active healthy eaters pattern group vs active sleepers, non-sedentary, unhealthy eaters pattern group	0.58 (−4.97, 6.14)	0.83	2.72 (−2.98, 8.42)	0.34	1.78 (−3.41, 6.97)	0.50	1.71 (−2.74, 6.16)	0.45
LPA^a^	Active healthy eaters pattern group vs unhealthy pattern group	0.48 (−4.80, 5.75)	0.86	1.09 (−4.69, 6.86)	0.71	0.11 (−4.62, 4.84)	0.96	0.54 (−3.80, 4.88)	0.81
	Active non-sedentary, unhealthy eaters pattern group vs unhealthy pattern group	0.69 (−3.87, 5.25)	0.76	−1.03 (−5.98, 3.92)	0.68	−2.42 (−7.21, 2.37)	0.32	−0.94 (−4.86, 2.97)	0.63
	Active healthy eaters pattern group vs active non-sedentary, unhealthy eaters pattern group	0.21 (−6.40, 6.82)	0.95	−2.12 (−8.35, 4.12)	0.50	−2.52 (−7.88, 2.83)	0.35	−1.48 (−6.55, 3.59)	0.56
PCA^b^	‘Mixed’ active, high sleep, non-sedentary, unhealthy eating pattern score	0.03 (−1.72, 1.77)	0.98	1.80 (0.13, 3.47)	**0.035**	0.31 (−1.49, 2.11)	0.73	0.71 (−0.69, 2.11)	0.32
	‘Healthy’, active healthy eating pattern score	0.07 (−1.59, 1.73)	0.93	0.03 (−1.85, 1.92)	0.97	−0.31 (−1.83, 1.22)	0.69	−0.07 (−1.48, 1.34)	0.92
	‘Unhealthy’, low sleep, sedentary, high snacks pattern score	−1.24 (−3.05, 0.57)	0.17	−1.64 (−3.83, 0.56)	0.14	−0.84 (−2.75, 1.08)	0.39	−1.24 (−2.91, 0.44)	0.15
	‘Mixed’ inactive, sedentary, high sleep pattern score	−1.37 (−3.46, 0.71)	0.19	0.01 (−2.34, 2.36)	0.99	−0.23 (−2.49, 2.04)	0.84	−0.53 (−2.51, 1.46)	0.60

CI, confidence interval; CA, cluster analysis; LPA, latent profile analysis; PCA, principal component analysis.

Bold values indicate significant *P*-values.

a Pairwise comparisons for patterns derived using CA and LPA.

b Patterns identified by PCA comprise behavioural scores for all sample members, patterns from CA and LPA comprise groups of individuals.

Associations between behavioural patterns and academic outcomes are presented in Table 5. There were no differences in academic outcomes between pattern groups derived using CA. In contrast, for LPA, children in the healthy ‘active healthy eaters’ pattern group had lower estimated average scores (36.9 to 38.3 units lower) for numeracy and grammar compared with children in the unhealthy pattern group. Children in the mixed ‘active, non-sedentary, unhealthy eaters’ pattern group had lower estimated average scores (22.9 to 28.5 units lower) for reading, spelling and grammar compared with children in the unhealthy pattern group. For PCA: (i) the mixed ‘active, high sleep, non-sedentary, unhealthy eating’ pattern score was associated with lower spelling and grammar scores (9.1 to 11 units lower); (ii) the healthy ‘active healthy eating’ pattern score was associated with lower scores (7.1 to 9.7 units lower) for all academic domains except writing; (iii) the unhealthy ‘low sleep, sedentary, high snacks’ pattern score was associated with lower scores (6.5 to 11 units lower) for all domains except numeracy; and (iv) the mixed ‘inactive, sedentary, high sleep’ pattern score was associated with higher reading scores (11.7 units higher).

**Table 5:** Associations of behavioural patterns with academic performance

Method	Behaviour pattern groups (CA/LPA)/scores (PCA)^b^	Reading		Writing		Spelling		Numeracy		Grammar	
		β (95% CI)	*P*-value	β (95% CI)	*P*-value	β (95% CI)	*P*-value	β (95% CI)	*P*-value	β (95% CI)	*P*-value
CA^a^	Active healthy eaters pattern group vs unhealthy pattern group	−17.9 (−42.8, 7.0)	0.16	−1.4 (−19.0, 16.1)	0.87	−17.5 (−41.4, 6.4)	0.15	−23.1 (−46.6, 0.3)	0.05	−23.1 (−51.3, 5.2)	0.11
	Active sleepers, non-sedentary, unhealthy eaters pattern group vs unhealthy pattern group	−1.7 (−22.1, 18.7)	0.87	12.3 (−2.6, 27.1)	0.11	−7.7 (−26.7, 11.2)	0.42	−0.6 (−20.3, 19.2)	0.96	1.1 (−20.6, 22.9)	0.92
	Active healthy eaters pattern group vs active sleepers, non-sedentary, unhealthy eaters pattern group	16.22 (−8.9, 41.3)	0.20	13.7 (−1.0, 28.4)	0.068	9.7 (−11.8, 31.2)	0.37	22.6 (2.7, 42.5)	0.027	24.2 (−0.7, 49.0)	0.057
LPA^a^	Active healthy eaters pattern group vs unhealthy pattern group	−20.7 (−42.9, 1.4)	0.067	−12.3 (−27.0, 2.4)	0.10	−20.4 (−41.9, 1.1)	0.063	−38.3 (−60.4, −16.2)	**0.001**	−36.9 (−61.7, −12.0)	**0.004**
	Active non-sedentary, unhealthy eaters pattern group vs unhealthy pattern group	−22.9 (−43.1, −2.7)	**0.026**	−5.4 (−20.7, 9.8)	0.48	−22.9 (−42.9, −2.9)	**0.025**	−18.1 (−38.1, 2.0)	0.077	−28.5 (−48.3, −8.6)	**0.005**
	Active healthy eaters pattern group vs active non-sedentary, unhealthy eaters pattern group	−2.2 (−29.5, 25.2)	0.88	6.8 (−11.9, 25.6)	0.47	−2.5 (−27.0, 22.0)	0.84	20.2 (−0.26, 40.7)	0.053	8.4 (−18.6, 35.4)	0.539
PCA^b^	‘Mixed’ active, high sleep, non-sedentary, unhealthy eating pattern score	−4.0 (−11.3, 3.3)	0.28	−3.6 (−8.3, 1.0)	0.12	−11.0 (−17.5, −4.43)	**0.001**	−4.5 (−10.4, 1.5)	0.14	−9.1 (−15.8, −2.5)	**0.008**
	‘Healthy’, active healthy eating pattern score	−7.6 (−14.1, −1.1)	**0.023**	−3.3 (−7.6, 0.9)	0.13	−7.1 (−13.7, −0.5)	**0.034**	−9.6 (−15.9, −3.4)	**0.003**	−9.7 (−17.7, −1.7)	**0.018**
	‘Unhealthy’, low sleep, sedentary, high snacks pattern score	−8.6 (−15.4, −1.7)	**0.015**	−6.5 (−12.1, −1.0)	**0.021**	−6.7 (−13.0, −0.4)	**0.039**	−5.5 (−11.8, 0.9)	0.092	−11.0 (−18.0, −4.1)	**0.002**
	‘Mixed’ inactive, sedentary, high sleep pattern score	11.7 (2.9, 20.6)	**0.010**	−2.7 (−8.5, 3.0)	0.35	1.1 (−7.8, 10.0)	0.81	6.9 (−0.9, 14.7)	0.083	11.7 (−0.1, 23.5)	0.052

CI, confidence interval; CA, cluster analysis; LPA, latent profile analysis; PCA, principal component analysis.

Bold values indicate significant *P*-values.

a Pairwise comparisons for patterns derived using CA and LPA.

b Patterns identified by PCA comprise behavioural scores for all sample members, patterns from CA and LPA comprise groups of individuals.

## Discussion

This is the first study to assess comparisons of associations between patterns derived from four behaviour domains using CA, LPA and PCA, and three outcomes, in children aged 6–8 years. This study compared associations between patterns derived using three different techniques and three childhood outcomes in a single dataset. CA and LPA derived three pattern groups each, whereas PCA identified four pattern scores. Associations between behavioural patterns and three outcomes differed by the three statistical approaches. The discord in these associations imply that the choice of method to derive patterns can influence subsequent associations with childhood outcomes.

Given that no prior studies have compared associations between patterns and outcomes using the three methods within a single study in children, the study findings were not directly comparable to previous work. Pattern groups derived using CA and LPA reported no differences in adiposity outcomes. However, evidence of associations with adiposity outcomes were observed for the unhealthy and mixed pattern scores from PCA. Despite differing associations observed across methods, the PCA results were consistent with findings from previous studies. The unhealthy and mixed pattern scores were associated with higher and lower adiposity risk, respectively. Associations of the unhealthy pattern scores with adiposity were similar to previous studies reporting increased risk of adiposity with patterns comprising high sedentary behaviour and snacking scores derived using PCA.^24–26^ These studies, however, did not investigate sleep in their patterns. Increased monitoring and tailored intervention strategies are warranted for children displaying unhealthy patterns, to reduce the obesity burden. The mixed inactive sedentary sleepers pattern has not been reported in previous studies and therefore evidence of associations with lower adiposity risk is novel. This pattern, despite being mixed, suggests that the healthy behaviour (high sleep duration) in this pattern appears to drive the association observed.^27^ Further investigation is warranted to assess mixed patterns with both unhealthy and healthy behaviours in relation to adiposity. In contrast to no associations between patterns from CA and LPA and adiposity, other studies deriving similar patterns have reported evidence of associations. Healthy patterns (healthy diets, high physical activity and/or low screen time)^28–30^ derived using CA were associated with lower adiposity risk. The healthy patterns from these studies all comprised low sedentary behaviour and might contribute to the differences in findings from our study. It could be that within patterns, low sedentary behaviour is a key driver along with a healthy diet and high physical activity to promote lower adiposity in children.

For HRQoL, children in the mixed pattern group from CA (active sleepers, non-sedentary, unhealthy eaters) had higher emotional, social and psychosocial functioning than those in the unhealthy pattern group. No evidence of differences in pattern groups derived using LPA were observed for HRQoL. The mixed pattern score from PCA (active sleepers, non-sedentary, unhealthy eaters) was associated with increased social functioning. Associations between mixed behavioural patterns from CA and PCA and HRQoL appear to be consistent for social functioning. Both mixed patterns were characterized by unhealthy diets, high physical activity, and sleep duration, and were non-sedentary. This is in line with evidence from analysis that assessed individual behaviours, except diet where higher HRQoL was associated with high physical activity and sleep duration, healthy diets and lower sedentary behaviour.^31^ Despite both mixed patterns derived from CA and PCA consisting of an unhealthy diet, this did not negate the beneficial effects of other behaviours on HROoL. It could be that high physical activity and sleep duration and lower sedentary behaviour are the main drivers towards HRQoL, and diet only secondary. Only one study^31^ investigating three behaviour domains (excluding sleep) using cluster analysis explored associations with HRQoL. Their mixed pattern group displayed highest scores for HRQoL; however, this pattern comprised low screen, moderate physical activity and sedentary behaviour and a healthy diet.

Associations with academic performance revealed that some pattern group differences from LPA and patterns identified from PCA were associated with lower academic scores. CA had no evidence of differences between pattern groups, whereas children in LPA’s healthy and mixed patterns had lower academic scores compared with those in the unhealthy pattern. All PCA patterns were associated with lower academic scores, except the mixed pattern (inactive sedentary sleepers) being associated with higher reading scores. Some sedentary behaviours, such as reading or homework, have been positively associated with higher academic performance.^32^ The inactive sedentary sleepers pattern was the only pattern from PCA (additionally not observed in any patterns from CA and LPA) characterized by high quiet play (apart from high sedentary behaviour) and could possibly account for why higher scores for reading and lower scores for all other academic domains across the three methods were observed. It could be that these sedentary behaviours are possible drivers within patterns when evaluating associations with academic performance. Our study findings are novel, as no other studies have investigated associations of patterns derived from four behaviour domains with academic performance. Only one study^32^ investigated patterns of physical activity, sedentary behaviour and diet using CA. They identified that children displaying unhealthy patterns (low physical activity and high sedentary behaviour) had higher academic performance than children displaying mixed patterns (high screen time, moderate PA and unhealthy diets). Although the evidence for individual behaviours^32–34^ suggests that healthier diets, more physical activity and sleep duration, low screen time but more time spent in reading/homework were associated with better academic performance, the evidence from patterns appear to refute this. It is possible that combined behavioural effects on academic performance may be less meaningful as compared with, for example obesity development, as they are primarily energy balance behaviours and not primary determinants of academic performance. It is also plausible that realistically, higher time spent performing other behaviours (healthy or unhealthy) would leave children less time to spend on academic activities.

Given the three methods use differing statistical algorithms, variations in associations reported with childhood outcomes were expected. Differences in associations reported could first be attributed to the discrepant number of patterns across the three methods. Second, the resultant patterns being continuous and categorical for PCA and CA/LPA, respectively, could explain some differences. PCA provides scores on all patterns for all individuals in a sample, whereas CA and LPA divide the whole sample into exclusive groups displaying similar patterns. Smaller samples within pattern groups from CA and LPA could have lacked power to detect associations. However these were observed only for adiposity measures, whereas some evidence of associations for HRQoL and academics was still observed. It is possible that the associations reported might be influenced not only by the method used to derive patterns but also by the childhood outcomes assessed and the measures used to capture them. This would not have stood out in our findings if we had assessed a single health outcome, as the findings varied not only across patterns but also across the outcomes themselves. The distribution of outcome data could potentially be another contributing factor to the differences observed across methods. The latent profile model used, being conservative, could have resulted in smaller standard errors compared with using an improved three-step approach,^35^ subsequently influencing associations observed between the patterns derived and health outcomes. Despite the differences observed across patterns from the three methods, the direction of the associations observed across methods were the same, which provides some confidence in the underlying associations. An advantage of using PCA over the other two methods is the avoidance of classification error, as all sample members have scores for the patterns identified. Additionally, the patterns from CA and LPA being categorical and the differences in the test of associations (independent vs group differences) between the methods, could partly explain why more evidence of associations were observed with PCA.

A major strength of this study is the comparison of associations between patterns from three techniques (CA, LPA and PCA) and three outcomes (adiposity, HRQoL and academic performance) in school-aged children. Previous studies have examined comparisons of one or two behaviour domain and no studies have investigated this age group, making our study findings novel. The current study included four behaviour domains to identify patterns providing a more comprehensive picture of the associations with outcomes compared with previous studies examining single behaviour domains.

Limitations of the study include the predominant use of survey data, leading to potential bias in the data captured, potentially affecting patterns derived and subsequent associations reported. However, these measures demonstrated sufficient reliability and were more feasible for large population studies. Furthermore, the inclusion of accelerometer data and objectively measured anthropometric measures accounts for the inclusion of objective data and increases confidence in the patterns derived and associations reported. The study being cross-sectional limited the inference of causal relationships. A larger sample would have been beneficial in having more power to detect pattern group differences from CA/LPA with childhood outcomes.

## Conclusion

This study compared associations of patterns derived from four behaviour domains, using three data reduction techniques (CA, LPA and PCA) with three outcomes (adiposity, HRQoL, academic performance) in school-aged children. Associations differed by the three methods, but some alignment in results was observed across methods. Not all dominant individual behaviours within patterns were drivers of the associations observed across methods and outcomes. Associations of behavioural patterns with childhood outcomes appeared to be different from previous studies that assessed individual behaviours, suggesting that the combined influence of health behaviours can be different and are not always consistent with their individual effects. These findings suggest that the choice of method used to derive lifestyle patterns has potential to influence subsequent associations observed with childhood outcomes. Additionally, direct comparability of associations across studies using differing methods may not be appropriate. It is important for future studies to thoroughly assess and justify their choice of method for future pattern derivation studies to best answer their research question.

## Ethics approval

The Deakin University Human Research Ethics Committee (EC 291–2007), the Department of Education and Early Childhood Development (2011–001008) and the Catholic Education Office (1714) granted ethics approval for the HAPPY Study. Written informed consent to participate was obtained from parents.

## Supplementary Material

dyac142_Supplementary_Data

## Data Availability

The data underlying this article cannot be shared publicly due to ethical restrictions related to the consent provided by participants during the study period. Upon approval from Deakin University Human Research Ethics Committee, an ethically compliant dataset may be made available by the corresponding author.
