# Supplementary material for: Differing associations with childhood outcomes using behavioural patterns derived from three data reduction techniques
Source: Int J Epidemiol. 2022 Jul 13;52(2):577–88. doi: 10.1093/ije/dyac142 (PMC10114100; doi:10.1093/ije/dyac142)
Supplement: dyac142_Supplementary_Data [file dyac142_supplementary_data.docx]

Table S1: Distribution of children in pattern groups across Latent profile analysis and Cluster analysis

|  | | **Latent profile analysis** | | |  |
| --- | --- | --- | --- | --- | --- |
|  |  | Unhealthy | Active healthy eaters | Active non-sedentary unhealthy eaters | Total |
| **Cluster analysis** | Unhealthy | 124 | 2 | 7 | **133** |
|  | Active healthy eaters | 17 | 78 | 7 | **102** |
|  | Active sleepers, non-sedentary unhealthy eaters | 68 | 2 | 127 | **197** |
|  | Total | **209** | **82** | **141** | **432** |

Table S2: Kappa agreement between pattern groups from latent class analysis and cluster analysis

| Agreement | Expected Agreement | Kappa | Std. Err. | Z | Prob>Z |
| --- | --- | --- | --- | --- | --- |
| 76.16% | 34.26% | 0.6373 | 0.0333 | 19.16 | 0 |

Table S3: Sample characteristics of latent profile and cluster analysis pattern groups

| Variable | Latent Profile Analysis | | | | Cluster Analysis | | | |
| --- | --- | --- | --- | --- | --- | --- | --- | --- |
|  | Unhealthy | Active healthy eaters | Active non-sedentary unhealthy eaters | Unhealthy | | Active healthy eaters | Active sleepers non-sedentary unhealthy eaters |  |
|  | Mean ± SD | Mean ± SD | Mean ± SD | Mean ± SD | | Mean ± SD | Mean ± SD |  |
| Child age (years) | 7.7 ± 0.7 | 7.8 ± 0.6 | 7.4 ± 0.7 | 7.8 ± 0.7 | | 7.7 ± 0.6 | 7.4 ± 0.7 |  |
| Sex (n)  Male  Female | 102  107 | 45  37 | 97  44 | 69  64 | | 53  49 | 122  75 |  |
| Parent education (n)  Below university  University and above | 70  139 | 32  50 | 42  99 | 53  80 | | 37  65 | 54  143 |  |
| Adiposity measures  BMI (kg/m^2^)  Waist circumference (cm) | 16.6 ± 2.0  58.7 ± 6.8 | 16.5 ± 1.6  59.0 ± 5.0 | 16.5 ± 1.6  58.1 ± 5.2 | 16.9 ± 2.3  59.6 ± 7.3 | | 16.6 ± 1.5  59.0 ± 5.1 | 16.3 ± 1.5  57.6 ± 5.3 |  |
| Health related Quality of life*  Emotional functioning score  Social functioning score  School functioning score  Psychosocial functioning score | 71.9 ± 13.7  83.0 ± 14.9  82.5 ± 15.0  79.2 ± 11.6 | 72.1 ± 15.7  83.7 ± 15.7  81.7 ± 15.3  79.2 ± 12.0 | 73.2 ± 16.9  82.7 ± 17.7  79.1 ± 16.0  78.3 ± 14.4 | 67.6 ± 13.7  77.8 ± 18.3  78.8 ± 17.6  74.8 ± 14.1 | | 73.2 ± 15.1  82.8 ± 15.6  80.9 ± 15.6  79.0 ± 11.8 | 74.3 ± 15.5  85.9 ± 14.2  82.6 ± 13.8  81.0 ± 11.7 |  |
| NAPLAN scores*  Reading  Writing  Spelling  Numeracy  Grammar | 491.3 ± 78.0  451.9 ± 52.8  453.9 ± 74.5  462.2 ± 82.0  502.0 ± 94.0 | 468.9 ± 89.1  438.1 ± 50.9  432.7 ± 79.2  423.2 ± 68.1  462.0 ± 93.0 | 468.5 ± 84.9  439.4 ± 67.7  429.1 ± 75.9  449.5 ± 72.7  470.5 ± 86.2 | 484.0 ± 77.2  442.6 ± 67.0  450.3 ± 75.5  453.8 ± 79.4  489.1 ± 93.9 | | 466.6 ± 89.9  440.4 ± 49.4  432.7 ± 76.1  431.8 ± 72.3  466.5 ± 89.6 | 483.4 ± 82.8  449.3 ± 56.0  441.0 ± 77.3  458.5 ± 78.1  490.1 ± 92.8 |  |

Abbreviations: BMI = body mass index, SD = standard deviation, NAPLAN = National Assessment Program - Literacy and Numeracy Testing

*Higher scores indicate higher health related quality of life and academic performance
